# Effect of intranasal esketamine on cognitive functioning in healthy participants: a randomized, double-blind, placebo-controlled study

**DOI:** 10.1007/s00213-018-4828-5

**Published:** 2018-02-01

**Authors:** Randall L. Morrison, Maggie Fedgchin, Jaskaran Singh, Joop Van Gerven, Rob Zuiker, Kyoung Soo Lim, Peter van der Ark, Ewa Wajs, Liwen Xi, Peter Zannikos, Wayne C. Drevets

**Affiliations:** 1 Neuroscience Integrative Solutions, 1125 Trenton-Harbourton Road, Titusville, NJ 08560 USA; 2 Janssen Research & Development, LLC, Titusville, NJ USA; 3 Centre for Human Drug Research, Leiden, The Netherlands; 4 CHA University School of Medicine and CHA Budang Medical Center, Seongnam, South Korea; 5 Janssen Research & Development, a Division of Janssen Pharmaceutica NV, Beerse, Belgium

**Keywords:** Cognitive functioning, Cogstate^®^ computerized test battery, Intranasal esketamine, Treatment-resistant depression

## Abstract

**Background:**

The effect of intranasal esketamine on cognitive functioning in healthy participants is assessed in this study.

**Methods:**

Twenty-four participants (19–49 years) were randomized to one of two treatment sequences in which either esketamine 84 mg or placebo was intranasally administered in a double-blind, two-period crossover design. Primary measures included five tests of Cogstate^®^ computerized test battery assessed at 1 h predose and 40 min, 2, 4, and 6 h postdose. Secondary measures included the Mental Effort Scale, Karolinska Sleepiness Scale (KSS), and safety.

**Results:**

Esketamine was associated with significant cognitive performance impairment at 40 min postdose for all five Cogstate® tests (Detection *p* = 0.0011, Identification *p* = 0.0006, One-Card Learning *p* = 0.0040, One Back *p* = 0.0017, and Groton Maze Learning Test *p* < 0.0001) versus placebo. In contrast, performance on these tests did not differ significantly between esketamine and placebo at 2, 4, or 6 h postdose. Secondary outcomes indicated a significant, transient increase from baseline under esketamine versus placebo at 40 min postdose on the Mental Effort Scale and at 40 min and 2 h postdose on KSS (*p* < 0.0001 for both); however, no significant difference was observed on these outcomes between esketamine and placebo at later timepoints. The most commonly reported adverse events were dizziness (67%), nausea (37.5%), disturbance in attention (29.2%), and fatigue (29.2%); the majority were considered mild in severity.

**Conclusions:**

Esketamine was associated with cognitive performance decline, and greater effort was required to complete the test battery versus placebo at 40 min postdose, which returned to placebo-comparable levels by 2 h postdose. Trial registration: ClinicalTrials.gov identifier: NCT02094378

**Electronic supplementary material:**

The online version of this article (10.1007/s00213-018-4828-5) contains supplementary material, which is available to authorized users.

## Introduction

Major depressive disorder (MDD) is a leading cause of disability worldwide and the most common antecedent illness to suicide (WHO Fact Sheet, reviewed April 2016). Although many psychopharmacological agents are currently available for the treatment of MDD, a substantial proportion of patients with MDD are resistant to conventional monoaminergic antidepressants and more effective interventions are needed for treatment-resistant depression (TRD). Additionally, patients who do respond to currently approved antidepressants require up to 4–6 weeks to show any improvement (Machado-Vieira et al. 2009).

Ketamine, an N-methyl-D-aspartate (NMDA) receptor antagonist, has been reported to exert a rapid onset of antidepressant effect in patients with TRD (Newport et al. 2015). Intranasal ketamine has shown safety and efficacy as an anesthetic and analgesic agent (Weksler et al. 1993; Louon and Reddy 1994; Diaz 1997; Weber et al. 2003). Intravenous ketamine has shown impairing effects on cognitive performance parameters that typically peak following administration and resolve within a few minutes to up to 2 h after drug discontinuation (Mathew et al. 2010; Murrough et al. 2013a, b; Zarate et al. 2006).

Several studies have examined cognitive function in infrequent and frequent ketamine users (Narendran et al. 2005; Morgan and Curran 2006; Morgan et al. 2009). Overall, infrequent or recreational ketamine use does not appear to be associated with long-term cognitive impairment (Narendran et al. 2005). In contrast, frequent ketamine users (more than five times per week) exhibit impairments in both short- and long-term memory (Morgan and Curran 2006). Although dosages used have varied, the doses reported by frequent ketamine users in this study were much higher than the doses that have been reported in the literature to alleviate depression severity in TRD (Newport et al. 2015) and usually used in combination with other drugs. Memory impairments may be reversible when individuals stop using the drug, as they were not found in a group of 30 former ketamine users who had been abstinent for at least one year (Morgan et al. 2010).

Esketamine (JNJ54135419), the S-enantiomer of ketamine racemate, has three to four times higher affinity for NMDA receptors than the R-enantiomer (Himmelseher and Pfenninger 1998), allowing antidepressant efficacy at lower doses (Singh et al. 2016). The intranasal route of administration is generally more convenient than intravenous delivery and also circumvents the relatively poor bioavailability associated with the oral route of administration for ketamine and esketamine. The relatively rapid onset of action and increased bioavailability of the drug administered via the intranasal delivery route are attributable to the rich vasculature and relatively high systemic absorption of esketamine via the nasal mucosa (Andrade 2015). The absolute bioavailability of intranasal esketamine 20 mg and 25 mg in healthy participants ranged between 45 and 59% (Bitter 2011; Yanagihara et al. 2003).

In healthy participants, intranasal esketamine has been relatively well-tolerated (Bitter 2011). However, the potential effects of intranasal esketamine on cognitive functioning have not been studied previously. Here, we evaluate the magnitude and duration of effects on cognitive function of a single dose of intranasal esketamine 84 mg in healthy volunteers.

## Methods

### Study population

Healthy men and women aged 19 to 49 years, with a body mass index 18 to 30 kg/m^2^ and a body weight of no less than 45 kg, were recruited into the study. Participants were excluded if they had systolic blood pressure measurements less than 90 mmHg or greater than 140 mmHg, diastolic blood pressure higher than 90 mmHg, or clinically significant abnormalities on electrocardiogram. Participants were also excluded if they had clinically significant medical conditions (including primary sleep disorder), abnormal laboratory values or abnormal physical/nasal examination, current or prior diagnosis of psychosis/psychotic disorder, or performance greater than one standard deviation (SD) below the mean on any of the five tests of the Cogstate^®^ computerized test battery during the training session.

### Study design

This phase 1, double-blind (DB), randomized, placebo-controlled, two-period crossover study was conducted at a single center in the Netherlands from June 2014 to August 2014 (NCT02094378).

**Fig. 1:**
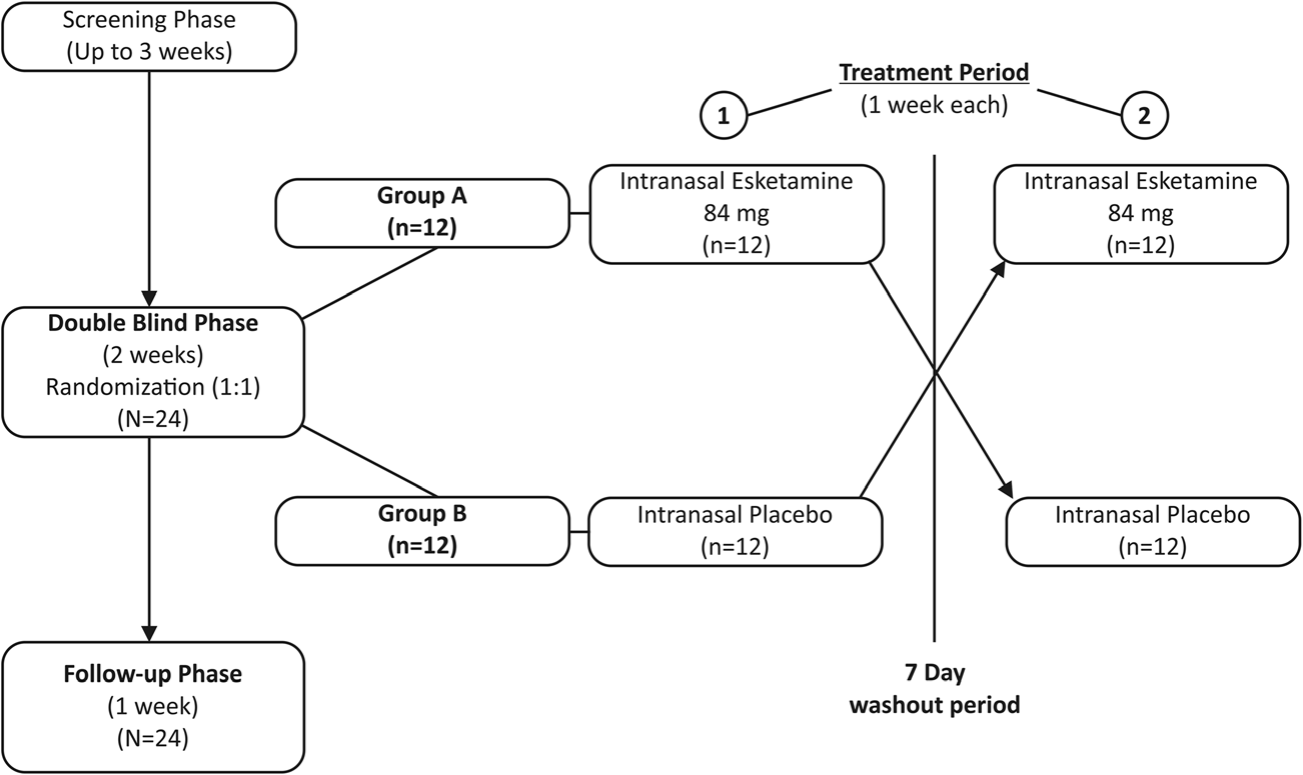
Study design and participant flow

The study consisted of three phases: screening phase (up to 3 weeks), DB treatment phase (2 weeks), and post-treatment (follow-up) phase (1 week). During the screening phase, evaluation of eligibility for participation included two training sessions on the Cogstate^®^ computerized test battery. The DB treatment phase consisted of two treatment periods (periods 1 and 2) separated by a washout interval of at least 7 days. On day 1 of study period 1, participants were randomized to one of two treatment sequences (treatment sequence 1: intranasal esketamine 84 mg followed by intranasal placebo; treatment sequence 2: intranasal placebo followed by intranasal esketamine 84 mg) in a two-way crossover design (Fig. 1). Randomization was based on a computer-generated randomization schedule prepared before the study by or under the supervision of the sponsor. The intranasal dose of esketamine 84 mg was selected because it produced a pharmacokinetic profile similar to that of an intravenously administered esketamine dose of 0.2 mg/kg, which showed antidepressant effects similar to higher doses of intravenous esketamine (Singh et al. 2016; Daly et al. 2017). The investigators, participants, and all study staff were kept blinded to the assigned treatment at randomization. Methylxanthine-containing products (chocolate bars, beverages, coffee, teas, colas, alcohol, etc) were prohibited from 24 h prior to administration of study medication.

### Assessments

#### Cognitive functioning measures

The primary endpoint was change in cognitive performance on each cognitive test from 1 h predose to each postdose timepoint on day 1 (40 min, 2 h, 4 h, and 6 h) and at 2-h intervals until participants returned to predose cognitive function for each treatment period. Using the Cogstate^®^ computerized test battery, the study evaluated multiple cognitive domains, including attention, visual and working memory, and executive functioning. The Cogstate^®^ computerized test battery consists of five tests/scores: Detection, Identification, One-Card Learning, One Back Memory, and Groton Maze Learning. The tests use playing card stimuli and a maze task, enabling use in multilingual/multicultural settings. Each test has been utilized in earlier drug studies, and the sensitivity of the Cogstate battery has been validated across repeated testing and cross-sectional research designs (Olver et al. 2008; Yoshida et al. 2012; McIntyre et al. 2014; Shiroma et al. 2014). The difference in each participant’s performance on any cognitive measure from predose was based on the Reliable Change Index (RCI), with an absolute value of RCI ≥ 1.96 considered to be a meaningful change from the predose score for a test. Participants whose performance on any cognitive test had not returned to predose performance level by 6 h postdose continued cognitive testing at 2-h intervals until all test scores had returned to predose levels. The criterion for continued testing was any cognitive test score that in comparison to the predose score fell in a range defined by the absolute value of |RCI| ≥ 1.96 (i.e., RCI ≤ − 1.96 or RCI ≥ 1.96). The secondary endpoints were the level of effort needed to complete the Cogstate^®^ computerized test battery and the level of sleepiness, as assessed by the change from baseline to each postdose timepoint on day 1 (40 min, 2 h, 4 h, and 6 h) for each treatment period using the Mental Effort Scale and the Karolinska Sleepiness Scale (KSS), respectively.

#### Safety and tolerability

Safety was evaluated by recording treatment-emergent adverse events (TEAEs), laboratory tests, vital signs, physical examinations, electrocardiogram monitoring, Columbia Suicide Severity Rating Scale (C-SSRS), Modified Observer’s Assessment of Alertness/Sedation (MOAA/S), Brief Psychiatric Rating Scale (BPRS+), and Clinician Administered Dissociative States Scale (CADSS). Additionally, local nasal tolerability was assessed by the nasal tolerability questionnaire and nasal examination.

### Statistical methods

#### Sample size determination

The sample size was prespecified to 24 participants. Assuming 80% of power and a two-sided significance level of 0.05, with a sample size of 24 participants, the study was able to detect the minimal detectable differences between esketamine and placebo in each cognitive test: Detection: 0.04, Identification: 0.04, One-Card Learning: 0.08, One Back: 0.15, and Groton Maze Learning Test: 12.87; assuming within-participant SDs 0.06, 0.07, 0.13, 0.25, and 21.5 for the aforementioned five tests obtained from the healthy control group (*n* = 120) in a study comparing the Cogstate^®^ battery and the Measurement and Treatment Research to Improve Cognition in Schizophrenia (MATRICS) battery (Pietrzak et al. 2009). A mixed-effect analysis of variance (ANOVA) model was used to assess the treatment difference in terms of five tests of the Cogstate^®^ Computerized Test Battery. The units for these tests are the mean of log10 transformed reaction times for correct responses (log 10 ms) on Detection and Identification tests, arcsine transformation of the proportion of correct responses for the One-Card Learning test, speed of performance (log 10 ms) for the One Back Test, and total number of errors committed for the Groton Maze Learning Test.

#### Analysis set

The intent-to-treat (ITT) or safety analysis set included all randomized participants who received at least one dose of study medication during the DB phase.

#### Statistical analyses

Statistical analyses were performed using SAS^®^ version 9.2. A mixed-effect analysis of variance (ANOVA) model was applied to each of the five tests of the Cogstate^®^ Computerized Test Battery to estimate treatment differences for each timepoint. The model included treatment, period, gender, and sequence as fixed effects and participants within sequence as a random effect. Baseline measures of scores were added as a covariate to the mixed-effect analysis of variance model. For this primary analysis (i.e., five Cogstate^®^ tests at 40 min), the Hochberg procedure was utilized to control the family-wise error rate. In addition, for assessing the learning effect, screening period versus period 1 predose and screening period versus period 2 predose were examined for the five Cogstate^®^ tests using paired *t* tests. To assess sex effects, a mixed-effect model was applied using repeated measures (MMRM) with baseline as a covariate, the period, sequence, time, sex and sex-by-time interaction as fixed effects, and the subject as a random effect for each of the five Cogstate^®^ tests. Mental Effort Scale and KSS were analyzed using the aforementioned mixed-effect model for the primary analysis. The incidence of adverse events was summarized by treatment group.

## Results

### Participants

All 24 enrolled and randomized participants completed the study. The demographics and baseline characteristics were comparable across the groups assigned to treatment sequence A versus B (Table 1).

**Table 1:** Demographic and baseline characteristics

	Treatment sequence 1^a^*n* = 12	Treatment sequence 2^b^*n* = 12
Age (years)		
Mean (SD)	23.7 (8.07)	27.0 (7.86)
Gender, *n* (%)		
Male	6 (50)	6 (50)
Race, *n* (%)		
White	11 (92)	11 (92)
Other	1 (8)	0
Black or African American	0	1 (8)
Ethnicity, *n* (%)		
Not Hispanic or Latino	10 (83)	12 (100)
Hispanic or Latino	2 (17)	0
BMI (kg/m^2^)		
Mean (SD)	22.7 (2.81)	24.4 (3.67)

*BMI* body mass index, *SD* standard deviation

^a^Intranasal esketamine 84 mg/intranasal placebo

^b^Intranasal placebo/intranasal esketamine 84 mg

### Cognitive function measures

#### Primary cognitive function measures

**Fig. 2:**
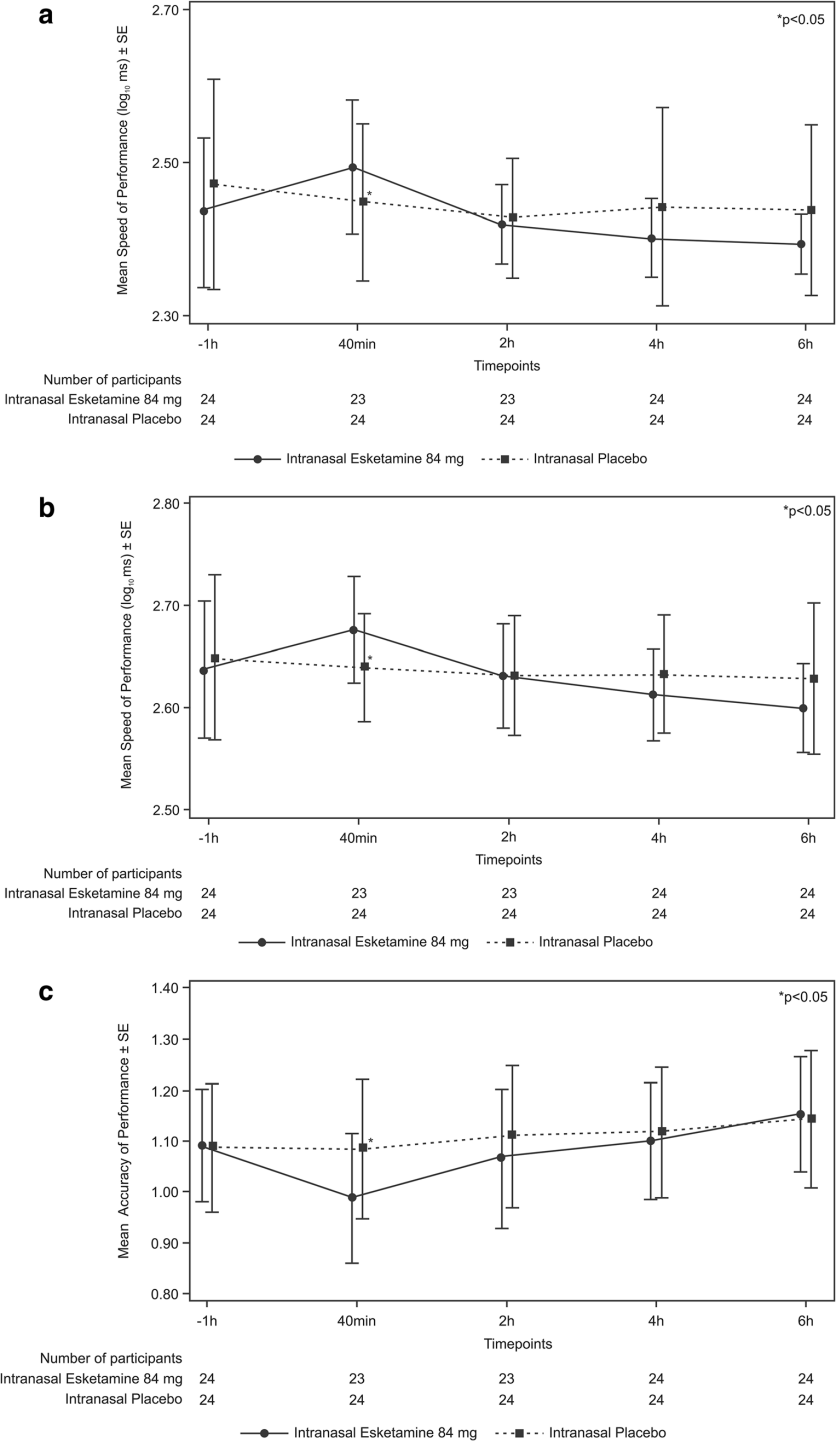

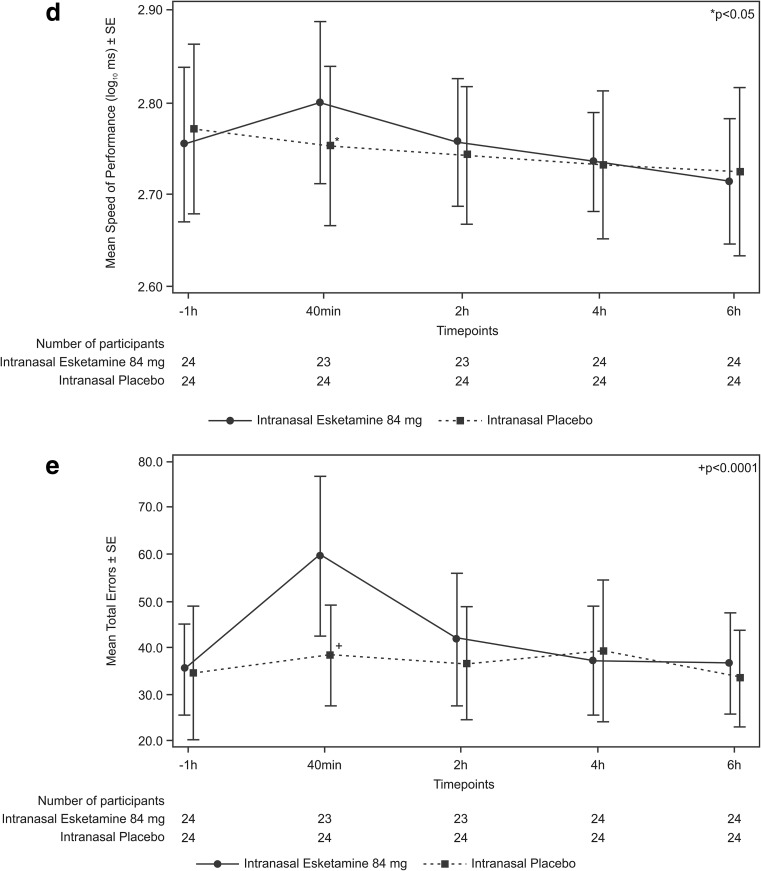
Cognitive function measures mean plots (± SE) for **a** Detection, **b** Identification, **c** One-Card Learning **d** One Back, **e** Groton Maze Learning Test (ITT Analysis Set)

Compared to predose assessments, cognitive performance declined to a greater extent under intranasal esketamine 84 mg than under placebo on each test at 40 min postdose (Table 2). At the 40 min postdose assessment, significant differences in the least squares (LS) mean (SE) values for the five Cogstate^®^ tests were noted for placebo versus esketamine (Fig. 2). The cognitive function in participants receiving esketamine returned to levels comparable to placebo by 2 h postdose. When comparing the two treatment groups, there was no significant difference between esketamine 84 mg and placebo in performance on any cognitive test at the 2-, 4-, or 6-h postdose assessments. A total of seven participants (esketamine: *n* = 4, placebo: *n* = 3) had |RCI| ≤ − 1.96 on at least one cognitive test at 6 h post-treatment, whereas a total of 25 participants (esketamine: *n* = 14, placebo: *n* = 11) met the criteria for continued testing of RCI ≥ 1.96 on at least one test 6 h post-treatment. Several participants continued to exhibit performance on a cognitive test that differed from predose performance through 10 h post esketamine or placebo, such that four who received esketamine and two who received placebo continued to perform below baseline (i.e., RCI < − 1.96), while ten who received esketamine and four who received placebo performed better than baseline (RCI ≥ 1.96; see Online Resource, supplementary Tables 1 and 2).

**Table 2:** Cognitive Functioning Tests: LS Means (SE) Over Time

	Placebo (N=24)	Esketamine 84 mg (N=23/24)^a^	*p* value^b^
Detection^c^ (Log10 ms)			
40 minutes^d^	2.44 (0.014)	2.51 (0.014)	0.0011
2 hours	2.42 (0.011)	2.43 (0.011)	0.8217
4 hours	2.43 (0.011)	2.41 (0.011)	0.3802
6 hours	2.43 (0.013)	2.40 (0.013)	0.1358
Identification^c^ (Log10 ms)			
40 minutes	2.64 (0.009)	2.68 (0.009)	0.0006
2 hours	2.63 (0.010)	2.63 (0.010)	0.7941
4 hours	2.63 (0.009)	2.62 (0.009)	0.2226
6 hours	2.63 (0.011)	2.60 (0.011)	0.1290
One-Card Learning (arcsine of proportion of correct responses)			
40 minutes	1.09 (0.021)	0.99 (0.021)	0.0040
2 hours	1.11 (0.021)	1.07 (0.021)	0.1483
4 hours	1.12 (0.021)	1.10 (0.021)	0.4465
6 hours	1.15 (0.017)	1.15 (0.017)	0.7450
One Back Memory^c^ (Log10 ms)			
40 minutes	2.75 (0.012)	2.80 (0.012)	0.0017
2 hours	2.74 (0.012)	2.76 (0.012)	0.1579
4 hours	2.73 (0.010)	2.74 (0.010)	0.3791
6 hours	2.72 (0.011)	2.72 (0.011)	0.9674
Groton Maze Learning Test (total numbers of errors)			
40 minutes	38.5 (2.45)	59.7 (2.51)	<0.0001
2 hours	36.7 (2.26)	41.7 (2.31)	0.1250
4 hours	39.6 (2.17)	37.1 (2.17)	0.2986
6 hours	33.6 (1.79)	36.8 (1.79)	0.1422

Detection - speed of performance (Log10 ms), Identification - speed of performance (Log10 ms), One-Card learning - accuracy of performance, One Back memory - speed of performance (Log10 ms), Groton Maze learning test - total numbers of errors, ms - milliseconds

^a^N=23 in esketamine groups for 40 minutes and 2 hours testing periods, N=24 for 4 and 6 hours testing periods

^b^*p* values (2-sided with level of significance of 5%) are based on the mixed-effect model with baseline score as a covariate, and treatment, period, gender and sequence as fixed effects, and subject within sequence as a random effect

^c^For these timed tests, higher scores reflect poorer performance

^d^All times listed (40 minutes, 2, 4, and 6 hours) reflect the times following dosing at which the testing was performed

In order to evaluate potential learning effects, subjects’ performance on each test was evaluated across screening period versus period 1 predose and screening period versus period 2 predose. There was no significant difference in performance on cognitive tests between screening period versus period 1 predose (Table 3). There were differences for three tests—Detection (simple reaction time task appears to be longer at predose period 2 than that at screening period; LS mean difference = 0.089 and *p* = 0.006 < 0.05), Groton Maze Learning (total number of errors appears to be smaller at predose period 2 than that at screening period; LS mean difference = − 5.500 and *p* = 0.010 < 0.05), and Identification (choice reaction time paradigm appears to be longer at predose period 2 than that at screening period; LS mean difference = 0.065 and *p* = 0.003 < 0.05) between the screening period and period 2 pre-dose (Table 3). There were significant differences on performance at the screening period versus period 2 predose for Detection (*p* < 0.01), Groton Maze Learning (*p* = 0.01), and Identification (*p* < 0.01). With respect to gender, a significant difference was found in One-Card Learning at 40 min in the placebo group, but performance did not differ significantly between males and females in the other tests (Supplementary Table 3).

**Table 3:** Cognitive Functioning Tests: Comparisons of Screening vs Predoses (ITT Analysis Set)

Tests		
	Screening	Predose Period 1
N	24	24
Detection		
Mean (SD)	2.40 (0.051)	2.41 (0.062)
*p* value^a^		0.329
Identification		
Mean (SD)	2.61 (0.047)	2.61 (0.042)
*p* value		0.482
One-Card Learning		
Mean (SD)	1.09 (0.125)	1.08 (0.120)
*p* value		0.849
One Back Memory		
Mean (SD)	2.76 (0.072)	2.76 (0.086)
*p* value		0.697
Groton Maze Learning Test		
Mean (SD)	38.42 (9.117)	37.00 (14.200)
*p* value		0.567

*p* value – predose vs screening

Detection - speed of performance (Log10 ms), Identification - speed of performance (Log10 ms), One-Card learning - accuracy of performance, One Back memory - speed of performance (Log10 ms), Groton Maze learning test - total numbers of errors, ms – milliseconds

^a^*p* values (2-sided with level of significance of 5%) and CIs (2-sided) are based on paired t-test

### Secondary cognitive function measures

#### Mental Effort Scale

**Fig. 3:**
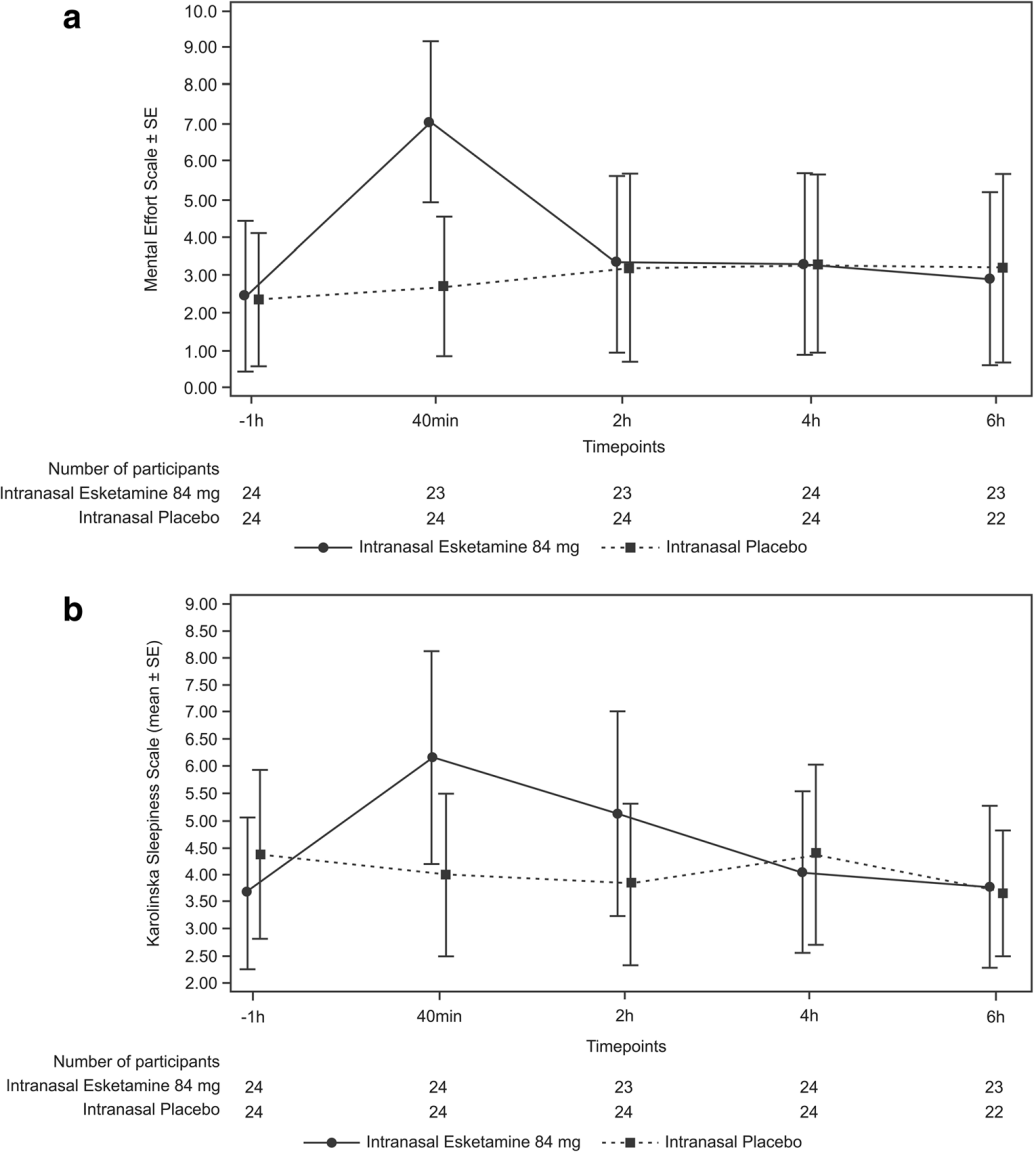
Secondary cognitive function measures mean plots (± SE) for **a** Mental Effort Scale and **b** Karolinska Sleepiness Scale (ITT Analysis Set)

Greater effort was required to complete the cognitive test battery after receiving esketamine versus placebo at the first postdose time point only (40 min postdose). Mental Effort Scale LS mean (SE) values at 40 min postdose were placebo: 2.74 (0.35) versus esketamine: 7.01 (0.358), *p* < 0.0001. Higher scores indicate greater mental effort required. The mental effort required to perform the cognitive tests returned to levels comparable to placebo by 2 h postdose (Fig. 3a), as the differences between esketamine versus placebo at the 2-, 4-, and 6-h postdose assessments were not statistically significant.

#### Karolinska Sleepiness Scale

Increased sleepiness was observed after esketamine administration compared to placebo, with significant differences between groups observed in the KSS at 40 min and 2 h postdose, returning to comparable levels as placebo by 4 h postdose. The KSS LS mean (SE) values (placebo versus esketamine) at 40 min postdose were 3.85 (0.344) versus 6.32 (0.344), *p* < 0.0001; at 2 h postdose: 3.53 (0.270) versus 5.46 (0.283), *p* < 0.0001; and at 4 h postdose: 4.14 (0.261) versus 4.27 (0.261), *p* = 0.72 (Fig. 3b).

### Safety

The most frequently reported TEAEs after intranasal esketamine administration included dizziness (*n* = 16, 67%), nausea (*n* = 9, 38%), disturbance in attention, fatigue and somnolence (*n* = 7, 29% each), and feeling abnormal (*n* = 6, 25%) (Table 4). The majority of TEAEs reported were rated as mild or moderate in intensity, and all TEAEs resolved at the end of the study. No deaths or other serious TEAEs, or TEAEs leading to discontinuation, occurred in this study.

**Table 4:** Treatment-emergent adverse events in at least 10% of participants in any treatment group (safety analysis set)

	Esketamine 84 mg *N* = 24	Placebo *N* = 24
Participants with 1 or more TEAEs	24 (100)	9 (38)
Dizziness	16 (67)	1 (4)
Headache	5 (21)	3 (13)
Disturbance in attention	7 (29)	0
Somnolence	6 (25)	1 (4)
Dysgeusia	3 (13)	1 (4)
Hypoaesthesia	4 (17)	0
Paraesthesia	3 (13)	1 (4)
Fatigue	7 (29)	0
Feeling abnormal	6 (25)	0
Feeling drunk	4 (17)	0
Feeling hot	4 (17)	0
Nausea	9 (38)	0
Vomiting	5 (21)	0
Vision blurred	4 (17)	0
Hallucination, visual	3 (13)	0

Values denoted as *n* (%). Percentages calculated with the number of participants in each group as denominator

*TEAE* treatment-emergent adverse events

**Fig. 4:**
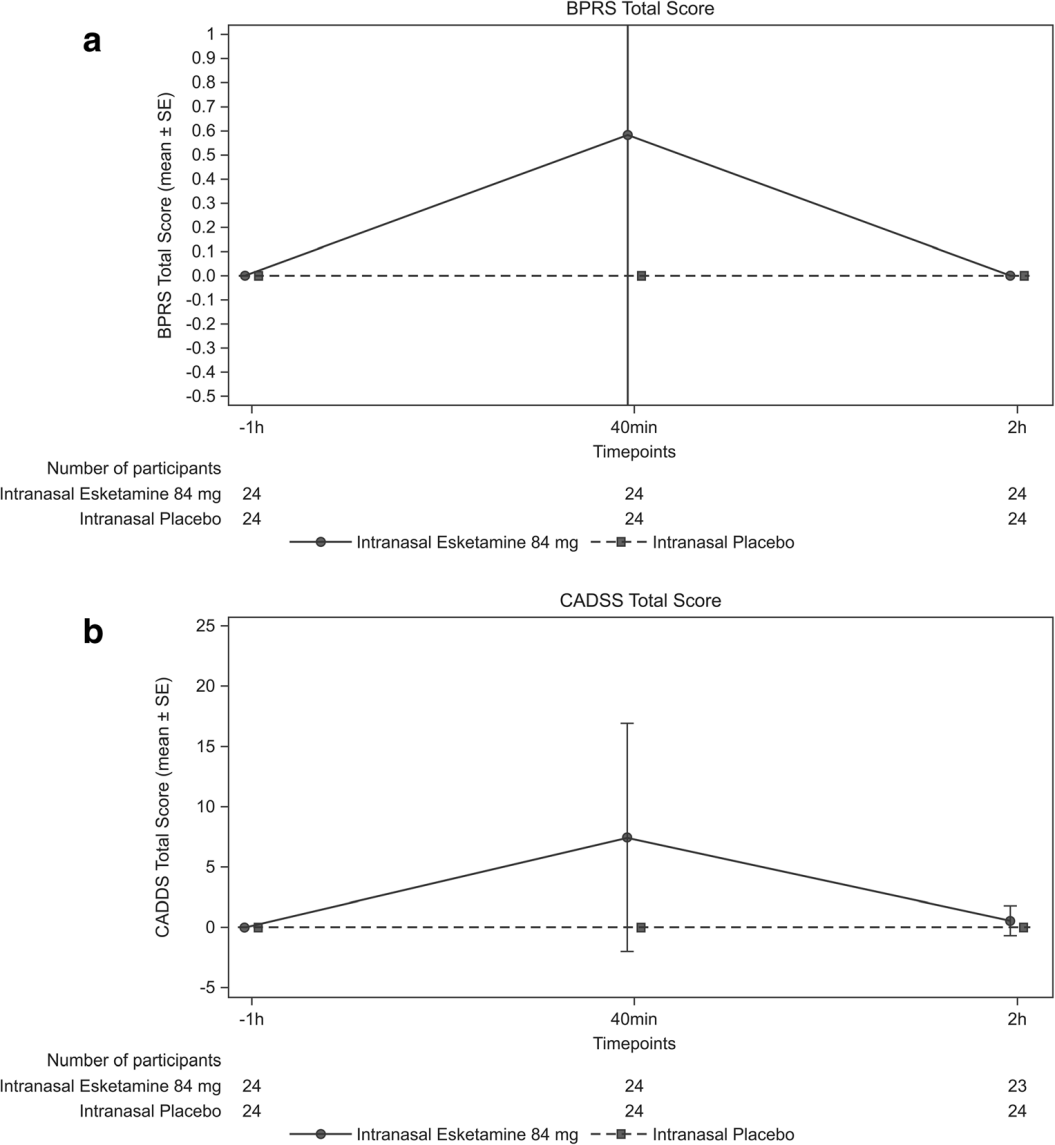
Total scores over time [mean (SD)] for **a** Brief Psychiatric Rating Scale and **b** Clinician-Administered Dissociative States Scale (Safety Analysis Set)

The dissociative symptoms assessed using the CADSS and treatment-emergent psychotic symptoms assessed using the BPRS+ were reported in participants receiving intranasal esketamine, with transient increases at 40 min postdose as compared to placebo, returning to baseline by 2 h postdose (Fig. 4a, b). Following esketamine administration, more participants demonstrated a transient increase in sedation, as assessed using the MOAA/S, compared to placebo (esketamine: *n* = 6; placebo: *n* = 1) through 4 h postdose. No dissociative or psychotic symptom and no change in sedation was reported in participants receiving intranasal placebo at any time point. No post-baseline change in suicidal ideation or behavior was evident on the CSSR-S in participants from either group. Overall, no clinically significant effect on the laboratory parameters, vital signs, or electrocardiogram parameters was observed.

## Discussion

This is the first clinical study to evaluate the effects of intranasal esketamine on cognitive functioning. In this exploratory study in healthy participants, administration of intranasal esketamine 84 mg was associated with a transient decline in cognitive function, manifested either as slower performance time or greater error rates at 40 min postdosing as compared to intranasal placebo, but performance returned to levels comparable to placebo by 2 h postdose. No significant difference between intranasal esketamine and placebo was observed at the 2-, 4-, and 6-h timepoints in the LS mean values for any of the five Cogstate^®^ tests. The transient reductions in cognitive function in participants receiving intranasal esketamine were associated with early postdose sedation, as assessed by the KSS and MOAA/S, and greater levels of effort required to complete the Cogstate^®^ computerized test battery, as assessed using the Mental Effort Scale. The increases in sleepiness (at 40 min and 2 h postdose) and the mental effort required at 40 min post esketamine administration returned to levels comparable to placebo by 4 and 2 h postdose for mental effort (Fig. 2).

The participant-level data were examined posthoc to assess whether any individuals showed outlying values suggestive of longer-lasting effects. This assessment revealed that some participants’ performance on cognitive tests had not returned to predose levels based on RCI criteria at 6 h post esketamine or longer; however, there were also participants whose cognitive test performance had not returned to predose levels at 6 h post placebo. Some participants continued to exhibit performance changes versus predose levels on one or more cognitive tests for up to 10 h postdose after esketamine or placebo, and these participant level data were most remarkable for the relatively larger number of participants whose performance appeared improved relative to baseline following esketamine (*n* = 10) versus after placebo (*n* = 4). While the potential contribution of fatigue or practice effects to these outlying values remains unclear, the apparent numerical difference between conditions in the numbers of participants showing improved performance at 10 h postdose merits further investigation, particularly in light of preclinical evidence in rodents that a single administration of ketamine alters synaptic plasticity in the hippocampus and medial prefrontal cortex (Kavalali and Monteggia 2012; Duman et al. 2016). Further discussion of participants meeting the RCI criterion for testing beyond 6 h postdose is provided in the Online Resource.

Performance on Detection, Identification, and Groton Maze Learning tests at period 2, 2 predose differed from screening, given that Detection and Identification scores declined at period 2 predose but Groton Maze improved, this is not likely a learning effect. The change from screening to period 2 predose was less than 1 SD (based on Cogstate test norms) for each test. The differences may reflect minor variability in cognitive status of some subjects (e.g., perhaps related to changes in sleep, or mild health ailments). It should also be considered that subjects’ period 1 experience in the study may have influenced anticipation/expectations about period 2. Several recent published reviews of studies of intravenous ketamine efficacy in MDD have highlighted that the side effects of ketamine may compromise blinding of subjects and investigators/raters, thus introducing potential bias (example, Newport et al. 2015). However, in contrast to self- or clinician ratings of mood/symptoms, which can be subjective, the present study measured cognitive performance via objective cognitive tests; all were computer administered and scored. The present study did not assess blinding efficacy, and while it cannot be assured that cognitive performance was unaffected by potential unblinding, as noted, there was no uniform directionality to the cognitive performance differences that were observed at period 2 predose versus screening. It will be important that procedures to minimize unblinding are optimized in efficacy trials of intranasal esketamine treatment for MDD, especially in regard to clinician ratings. The effect of intranasal esketamine on increased sleepiness assessed using KSS was more sustained relative to the effects on cognition, returning to near baseline levels by 4 h postdose.

Ketamine has been shown to exert a rapid onset of antidepressant effect in patients with TRD (Machado-Vieira et al. 2009; Murrough et al. 2013a, b; Newport et al. 2015; Yang et al. 2015). However, evidence shows that ketamine also is associated with acute perceptual and cognitive disturbances following drug administration in healthy participants as well as in patients with mood disorders (Krystal et al. 2005; Perry et al. 2007; Murrough et al. 2013a, b). Frequent abuse of high doses of ketamine can also lead to persistent neurocognitive impairment (Morgan and Curran 2006; Morgan et al. 2009).

In controlled studies of TRD patients, Murrough et al. previously reported circumscribed memory impairment immediately following a single ketamine dose (0.5 mg/kg) administered as a slow infusion over 40 min (Murrough et al. 2014). In contrast, two studies which explored the neurocognitive effects of up to six ketamine infusions in patients with TRD (with unipolar or bipolar depression) demonstrated no impairment (Diamond et al. 2014; Shiroma et al. 2014). Shiroma et al. (Shiroma et al. 2014) demonstrated that serial infusions of ketamine in TRD were not associated with cognitive decline over 4 weeks. This was consistent with the study by Diamond et al. (2014), where no memory deficits were noted after repeated ketamine administration, measured 4–7 days as well as 12 and 26 weeks after the final infusion. In the study by Shiroma et al. (2014) as well as in the recent study by Murrough et al. (2015), neurocognitive performance improved following treatment with intravenous ketamine. However, the studies were limited by small sample sizes, thus restricting a conclusive analysis of the relationship between cognitive effects and ketamine’s antidepressant activity. Also, only the report by Murrough et al. (2013a, b) assessed ketamine effects on cognitive function immediately after a single administration of intravenous ketamine. In the other studies, cognitive assessment was conducted days/weeks following a course of ketamine infusions; cognitive effects in the period 40–60 min post ketamine administration were not assessed. In the current study, the adverse cognitive effects experienced by these healthy participants were of short duration and resolved by 2 h postdose, returning to levels comparable to those obtained with placebo (Fig. 2). There has been no previous published report of the effects of intranasal esketamine on cognitive function in patients with MDD or on repetitive measurement to assess the time course and resolution of cognitive changes.

Our observations were substantiated using the Mental Effort Scale, an assessment of the level of effort needed to complete the test battery. Reductions in cognitive performance at 40 min postdose in participants receiving esketamine 84 mg were associated with a greater level of effort required to complete the test battery. The effect of intranasal esketamine on increased sleepiness, compared to placebo, as assessed by the KSS, was more sustained relative to the effects on cognition, returning to near baseline levels by 4 h postdose. However, repeated use of some drugs may lead to fewer side effects and increased tolerance over time, allowing patients to function at normal, placebo-comparable levels (Verster et al. 2015). It is yet to be seen if the effects of intranasal esketamine on reduced cognitive performance and increased sleepiness may attenuate with more consistent drug use in patients.

Consistent with previous observations obtained during intravenous infusion of esketamine in participants with TRD (Singh et al. 2016), most TEAEs observed following intranasal esketamine were rated mild or moderate in severity. All TEAEs resolved by the end of the study, and no severe or serious AE or deaths was reported. No treatment-emergent abnormal laboratory results or electrocardiogram values were reported in this study. The single intranasal dose of esketamine 84 mg showed no medically significant safety concerns in healthy participants. This safety profile was consistent with that observed in studies of TRD that involved intravenous ketamine or esketamine administration (aan het Rot et al. 2010; Singh et al. 2016).

The significance of our findings should be evaluated in the scope of study limitations. The study examined cognitive effects of only a single dose of intranasal esketamine, in a small sample of healthy volunteers. While the results contribute to the understanding of acute intranasal esketamine effects, this exploratory study is neither designed nor sufficient to enable conclusions about cognitive safety of intranasal esketamine. Rather, such conclusions will, at a minimum, require data from large clinical trials in patients with MDD in which the cognitive effects of intranasal esketamine are evaluated, including cognitive effects during and following both acute and maintenance treatment. A strength of the study design was the use of the Cogstate^®^ Battery, which has been validated against traditional neuropsychological tests (Maruff et al. 2009; Pietrzak et al. 2009) shown to have limited practice effects at brief test-retest intervals (Collie et al. 2003), and is sensitive to effects of various drugs on cognitive performance including ETOH and benzodiazepines (Maruff et al. 2005; Snyder et al. 2005).

Overall, the results demonstrate that a single dose of intranasal esketamine 84 mg was associated with a decline in cognitive function compared to placebo at 40 min postdose, which returned to levels comparable to placebo by 2 h postdose. The reductions in cognitive performance were associated with a greater level of effort required to complete the test battery. Sedation was longer lasting insofar as the drug versus placebo difference in KSS scores remained statistically significant at 2 h, but no significant difference in KSS scores was evident between conditions by 4 h postdose.

Finally, the observation that the number of participants showing improved performance at 10 h postdose relative to baseline was larger following esketamine than following placebo (10 versus 4, respectively) merits investigation in future studies designed to assess cognitive-enhancing as well as cognitive-impairing effects, particularly in light of preclinical evidence that ketamine administration increases synaptic plasticity in the hippocampus and medial prefrontal cortex in rodent stress models (Kavalali and Monteggia 2012, Duman et al. 2016). Further clinical testing also is needed to evaluate the potential differences in esketamine’s effects on cognition between acute administration versus repeated administration.

## Electronic supplementary material

ESM 1 (DOCX 29.9 kb)

ESM 2 (23.1 kb)

ESM 3 (22.2 kb)
