# Supplementary material for: Effect of intranasal esketamine on cognitive functioning in healthy participants: a randomized, double-blind, placebo-controlled study
Source: Psychopharmacology (Berl). 2018 Feb 1;235(4):1107–19. doi: 10.1007/s00213-018-4828-5 (PMC5869899; doi:10.1007/s00213-018-4828-5)
Supplement: Supplementary file 1 — (DOCX 29.9 kb) [file 213_2018_4828_MOESM1_ESM.docx]

**Supplementary Table 1. Participants in Treatment Sequence 1 with Reliable Change Index (RCI) < -1.96 and > 1.96 at timepoints ≥6 hours postdose**

|  |  | Treatment Sequence 1^a^ | | | | | | | | | | | | | |
| --- | --- | --- | --- | --- | --- | --- | --- | --- | --- | --- | --- | --- | --- | --- | --- |
|  |  | RCI < -1.96 | | | | | | | RCI > 1.96 | | | | | | |
|  |  | Esketamine 84 mg | | | Placebo | | | | Esketamine 84 mg | | | Placebo | | | |
| Participant* | Test | Postdose timepoints (hours) | | | | | | | | | | | | | |
|  |  | **6** | **8** | **10** | | **8** | **10** | **6** | | **8** | **10** | | **6** | **8** | **10** |
| **X1 (1004)** | **DETLMN** |  |  |  | | 1 |  |  | |  |  | |  |  |  |
|  | **OCLACC** |  |  |  | |  |  |  | |  |  | | 1 |  |  |
| **X2 (1005)** | **GMLTER** |  |  |  | |  |  |  | |  |  | | 1 |  |  |
| **X3 (1006)** | **DETLMN** |  |  |  | |  |  |  | |  |  | | 1 | 1 | 1 |
|  | **ONBLMN** |  |  |  | |  |  |  | |  |  | | 1 | 1 | 1 |
| **X4 (1010)** | **OCLACC** |  |  |  | |  |  | 1 | |  |  | |  |  |  |
| **X5 (1011)** | **GMLTER** |  |  |  | |  | 1 |  | |  |  | |  |  |  |
|  | **DETLMN** |  |  |  | |  |  |  | |  |  | | 1 | 1 | 1 |
|  | **IDNLMN** |  |  |  | |  |  |  | |  |  | | 1 | 1 |  |
|  | **OCLACC** |  |  |  | |  |  |  | |  |  | | 1 | 1 |  |
|  | **ONBLMN** |  |  |  | |  |  |  | |  |  | | 1 |  |  |
| **X6 (3001)** | **OCLACC** | 1 |  |  | |  |  |  | |  |  | |  |  |  |
|  | **GMLTER** |  |  |  | |  |  | 1 | |  |  | |  |  |  |
| **X7 (3003)** | **OCLACC** |  |  |  | |  |  | 1 | |  |  | |  |  |  |
|  | **ONBLMN** |  |  |  | |  |  |  | | 1 |  | |  |  |  |
| **X8 (3007)** | **GMLTER** |  |  | 1 | |  |  |  | |  |  | |  |  |  |
|  | **DETLMN** |  |  |  | |  |  |  | |  | 1 | |  |  |  |
|  | **IDNLMN** |  |  |  | |  |  |  | | 1 |  | |  |  |  |
|  | **OCLACC** |  |  |  | |  |  | 1 | | 1 |  | |  |  |  |
| **X9 (3008)** | **GMLTER** | 1 | 1 |  | | 1 |  |  | |  |  | |  |  |  |
|  | **OCLACC** |  | 1 |  | |  |  |  | |  |  | | 1 |  | 1 |
|  | **DETLMN** |  |  |  | |  |  |  | | 1 |  | | 1 |  |  |
|  | **IDNLMN** |  |  |  | |  |  |  | |  |  | | 1 | 1 | 1 |
| **X10 (3009)** | **DETLMN** |  |  |  | |  |  | 1 | | 1 | 1 | |  |  |  |
|  | **GMLTER** |  |  |  | |  |  |  | | 1 | 1 | |  |  |  |
| **X11 (3012)** | **GMLTER** |  |  | 1 | |  |  |  | |  |  | |  |  |  |
|  | **DETLMN** |  |  |  | |  |  |  | |  |  | | 1 | 1 | 1 |
|  | **IDNLMN** |  |  |  | |  |  |  | |  |  | |  | 1 | 1 |
|  | **OCLACC** |  |  |  | |  |  | 1 | | 1 | 1 | |  |  | 1 |
|  | **ONBLMN** |  |  |  | |  |  |  | |  |  | |  | 1 | 1 |
| ^a^Intranasal Esketamine 84 mg/ Intranasal Placebo  *Individual participants  RCI, Reliable Change Index; DETLMN, Detection; OCLACC, One-Card Learning; GMLTER, Groton Maze Learning Test; ONBLMN, One Back; IDNLMN, Identification. | | | | | | | | | | | | | | | |
