# Supplementary material for: Effect of intranasal esketamine on cognitive functioning in healthy participants: a randomized, double-blind, placebo-controlled study
Source: Psychopharmacology (Berl). 2018 Feb 1;235(4):1107–19. doi: 10.1007/s00213-018-4828-5 (PMC5869899; doi:10.1007/s00213-018-4828-5)
Supplement: Supplementary file 2 — (23.1 kb) [file 213_2018_4828_MOESM2_ESM.docx]

**Supplementary Table 2. Participants in Treatment Sequence 2 With Reliable Change Index (RCI) < -1.96 and > 1.96 at Timepoints ≥6 hours Postdose**

|  |  | Treatment Sequence 2^a^ | | | | | | | | | | | | |
| --- | --- | --- | --- | --- | --- | --- | --- | --- | --- | --- | --- | --- | --- | --- |
|  |  | RCI < -1.96 | | | | | | | | RCI > 1.96 | | | | |
|  |  | Placebo | | | Esketamine 84 mg | | | | | Placebo | | Esketamine 84 mg | | |
| Participant* | Test |  | Postdose timepoints (hours) | | | | | | | | | | | |
|  |  | **6** | **8** | **10** | | **6** | **8** | **10** | **6** | | **8** | **6** | **8** | **10** |
| **Y1 (1002)** | **IDNLMN** | 1 |  |  | |  |  |  |  | |  |  |  |  |
|  | **OCLACC** |  |  |  | |  |  |  | 1 | |  |  |  |  |
| **Y2 (1003)** | **ONBLMN** |  |  |  | |  |  |  |  | |  | 1 | 1 | 1 |
| **Y3 (1007)** | **IDNLMN** |  |  |  | |  |  |  |  | |  |  | 1 |  |
|  | **OCLACC** |  |  |  | |  |  |  |  | |  | 1 |  | 1 |
|  | **ONBLMN** |  |  |  | |  |  |  |  | |  |  | 1 |  |
| **Y4 (1008)** | **GMLTER** | 1 |  |  | |  |  |  |  | |  |  |  |  |
|  | **DETLMN** |  |  |  | |  |  |  |  | |  | 1 | 1 | 1 |
|  | **IDNLMN** |  |  |  | |  |  |  |  | |  | 1 | 1 | 1 |
|  | **OCLACC** |  |  |  | |  |  |  |  | |  |  | 1 | 1 |
|  | **ONBLMN** |  |  |  | |  |  |  |  | | 1 |  |  |  |
| **Y5 (1009)** | **GMLTER** |  | 1 |  | |  |  |  |  | |  |  |  |  |
|  | **OCLACC** |  |  |  | |  |  |  | 1 | | 1 | 1 |  |  |
| **Y6 (1012)** | **DETLMN** |  |  |  | |  |  |  | 1 | | 1 | 1 |  |  |
|  | **IDNLMN** |  |  |  | |  |  |  |  | |  | 1 |  |  |
|  | **ONBLMN** |  |  |  | |  |  |  |  | |  | 1 |  |  |
| **Y7 (3002)** | **GMLTER** |  |  |  | | 1 | 1 | 1 | 1 | |  |  |  |  |
|  | **OCLACC** |  | 1 |  | |  |  |  |  | |  |  |  |  |
|  | **DETLMN** |  |  |  | |  |  |  |  | |  | 1 | 1 | 1 |
|  | **ONBLMN** |  |  |  | |  |  |  |  | | 1 | 1 | 1 | 1 |
| **Y8 (3004)** | **DETLMN** |  |  |  | |  |  |  | 1 | | 1 | 1 | 1 | 1 |
|  | **IDNLMN** |  |  |  | |  |  |  |  | | 1 | 1 | 1 | 1 |
|  | **OCLACC** |  |  |  | |  |  |  |  | |  |  |  | 1 |
|  | **ONBLMN** |  |  |  | |  |  |  |  | | 1 | 1 | 1 | 1 |
| **Y9 (3005)** | **DETLMN** |  |  |  | |  |  |  |  | |  | 1 | 1 | 1 |
|  | **IDNLMN** |  |  |  | |  |  |  |  | |  |  |  | 1 |
|  | **ONBLMN** |  |  |  | |  |  |  |  | |  |  |  | 1 |
| **Y10 (3011)** | **DETLMN** |  |  |  | | 1 | 1 |  |  | |  |  |  |  |
|  | **GMLTER** | 1 | 1 |  | |  |  |  |  | |  |  |  |  |
|  | **OCLACC** |  | 1 | 1 | |  |  | 1 |  | |  |  |  |  |
|  | **ONBLMN** |  |  |  | |  |  |  |  | |  |  |  | 1 |
| ^a^Intranasal Placebo/ Intranasal Esketamine 84 mg.  *Individual participants  RCI, Reliable Change Index; DETLMN, Detection; OCLACC, One-Card Learning; GMLTER, Groton Maze Learning Test; ONBLMN, One Back; IDNLMN, Identification. | | | | | | | | | | | | | | |
