# Supplementary material for: Effect of intranasal esketamine on cognitive functioning in healthy participants: a randomized, double-blind, placebo-controlled study
Source: Psychopharmacology (Berl). 2018 Feb 1;235(4):1107–19. doi: 10.1007/s00213-018-4828-5 (PMC5869899; doi:10.1007/s00213-018-4828-5)
Supplement: Supplementary file 3 — (22.2 kb) [file 213_2018_4828_MOESM3_ESM.docx]

**Supplementary Table 3: Gender in Cognitive Functioning Tests: LS Means (SE) Over Time (ITT Analysis Set)**

|  | **Esketamine 84 mg** | | **Placebo** | |
| --- | --- | --- | --- | --- |
|  | **Male** | **Female** | **Male** | **Female** |
| **Detection** |  |  |  |  |
| N | 12 | 11 | 12 | 12 |
| 40 minutes | 2.47 (0.022) | 2.51 (0.022) | 2.44 (0.018) | 2.46 (0.018) |
| 2 hours | 2.41 (0.009) | 2.43 (0.01) | 2.41 (0.02) | 2.44 (0.02) |
| 4 hours | 2.41 (0.009) | 2.39 (0.009) | 2.43 (0.026) | 2.46 (0.026) |
| 6 hours | 2.4 (0.013) | 2.39 (0.013) | 2.41 (0.022) | 2.47 (0.022) |
| **Groton Maze learning test** |  |  |  |  |
| N | 12 | 11 | 12 | 12 |
| 40 minutes | 58.2 (4.39) | 61.03 (4.541) | 37.94 (2.041) | 38.9 (2.041) |
| 2 hours | 45.11 (3.712) | 37.95 (3.857) | 35.69 (2.475) | 37.56 (2.475) |
| 4 hours | 38.95 (2.8) | 35.32 (2.797) | 37.52 (3.217) | 41.23 (3.217) |
| 6 hours | 37.11 (2.677) | 36.32 (2.674) | 34.19 (2.303) | 32.81 (2.303) |
| **Identification** |  |  |  |  |
| N | 12 | 11 | 12 | 12 |
| 40 minutes | 2.67 (0.014) | 2.69 (0.015) | 2.65 (0.011) | 2.63 (0.011) |
| 2 hours | 2.64 (0.013) | 2.62 (0.013) | 2.63 (0.015) | 2.63 (0.015) |
| 4 hours | 2.62 (0.011) | 2.6 (0.011) | 2.64 (0.013) | 2.63 (0.013) |
| 6 hours | 2.61 (0.013) | 2.59 (0.013) | 2.62 (0.017) | 2.64 (0.017) |
| **One-back memory** |  |  |  |  |
| N | 12 | 11 | 12 | 12 |
| 40 minutes | 2.78 (0.019) | 2.82 (0.019) | 2.76 (0.015) | 2.74 (0.015) |
| 2 hours | 2.77 (0.015) | 2.75 (0.016) | 2.74 (0.02) | 2.74 (0.02) |
| 4 hours | 2.75 (0.011) | 2.72 (0.011) | 2.74 (0.017) | 2.73 (0.017) |
| 6 hours | 2.73 (0.016) | 2.7 (0.016) | 2.71 (0.016) | 2.74 (0.016) |
| **One-card learning** |  |  |  |  |
| N | 12 | 11 | 12 | 12 |
| 40 minutes | 0.98 (0.037) | 1.01 (0.038) | **1.04 (0.020)** | **1.14 (0.020)*** |
| 2 hours | 1.06 (0.035) | 1.08 (0.037) | 1.1 (0.023) | 1.13 (0.023) |
| 4 hours | 1.09 (0.033) | 1.11 (0.033) | 1.12 (0.026) | 1.12 (0.026) |
| 6 hours | 1.15 (0.024) | 1.16 (0.024) | 1.15 (0.027) | 1.14 (0.027) |
|  | | |  |  |

*P-value =0.0018

Detection - speed of performance (log10 ms), Groton Maze learning test - total errors, identification - speed of performance (log10 ms), one-back memory - speed of performance (log10 ms), one-card learning - accuracy of performance
